# Advances in metabolomics profiling of pediatric kidney diseases: A review 

**DOI:** 10.17305/bb.2024.10098

**Published:** 2024-10-01

**Authors:** Guoping Huang

**Affiliations:** 1 Department of Nephrology, Children’s Hospital, Zhejiang University School of Medicine, National Clinical Research Center for Child Health, Hangzhou, China

**Keywords:** Targeted metabolomics, untargeted metabolomics, multi-omics, pediatric renal diseases

## Abstract

Pediatric renal diseases encompass a diverse array of pathological conditions, often engendering enduring ramifications. Metabolomics, an emergent branch of omics sciences, endeavors to holistically delineate alterations in metabolite compositions through the amalgamation of sophisticated analytical chemistry techniques and robust statistical methodologies. Recent advancements in metabolomics research within the realm of pediatric nephrology have been substantial, offering promising avenues for the identification of robust biomarkers, the elaboration of novel therapeutic targets, and the intricate elucidation of molecular mechanisms. The present discourse aims to critically review the progress in metabolomics profiling pertinent to pediatric renal disorders over the previous 12 years.

## Introduction

Metabolomics is increasingly acknowledged as a dynamically evolving subfield of omics sciences, designed to offer a comprehensive characterization of metabolite variations—comprising small molecule chemicals extant in cellular environments and physiological fluids with molecular masses <1500 Da—utilizing advanced analytical chemistry techniques in synergy with statistical analyses [1, 2]. Positioned chronologically subsequent to genomics, transcriptomics, and proteomics, metabolomics aims for the quantitative delineation of metabolite variations across biological organisms. Notably, it encompasses an expansive range of over 3000 chemical classes and up to one million metabolites, as compared to the approximate 20,300 genes or the exceeding 620,000 protein species hitherto identified. This sheer volume renders metabolomics a particularly intricate subdivision of omics sciences [3]. Due to its proximal relationship to phenotypic outcomes, metabolomics offers invaluable insights into the underlying mechanisms governing physiological transformations [4].

Metabolites are categorized into primary and secondary constituents. Primary metabolites include sugars, nucleic acids, short peptides, lipids, and amino acids, which are generated via endogenous catabolic and anabolic processes [5]. Conversely, secondary metabolites are chiefly exogenous compounds, originating from environmental sources or dietary constituents, and encompassing substances such as food additives, pharmacological agents, microbial byproducts, and environmental pollutants [6–8]. The metabolome of an individual is subject to modulation by a multitude of internal and external variables, including, but not limited to, genetic predispositions, environmental exposures, circadian rhythms, nutritional intake, gender, and chronological age [9].

## The methodology of metabolomics

In juxtaposition to the human genome and proteome, the metabolome is categorically expansive, labyrinthine, and arduous to scrutinize [3]. Over successive decades, the methodological apparatuses underlying metabolomics have undergone substantive maturation, most notably through the integration of diverse analytical instruments. Global investigators predominantly deploy mass spectrometry (MS)-based or nuclear magnetic resonance (NMR)-based techniques to elucidate metabolomic complexities. MS-based methodologies employ a multifarious array of instrumental configurations, including but not limited to ion mobility spectrometry (IMS-MS), capillary electrophoresis (CE-MS), gas chromatography (GC-MS), and liquid chromatography (LC-MS) [10]. Within the purview of MS-based paradigms, the ionization of target molecules assumes paramount importance. Researchers delineate the physicochemical attributes of a specific compound by scrutinizing the mass-to-charge ratio (m/z) of an ionized molecular entity or its ionized fragments. These analyses are subsequently corroborated through comparative evaluations against extant reference mass spectra [3].

Contrastingly, NMR-based paradigms entail the detection of absorption bands or resonances characterized by unique radio frequencies, which emanate upon subjecting molecular entities or molecular conglomerates to an intensively potent magnetic field. Such molecular entities display distinctive NMR chemical shift patterns, contingent upon their chemical architectures and the spatial orientations of hydrogen atoms. Consequently, NMR obviates the imperative for molecular ionization or chromatographic demarcation [11].

**Figure 1.**
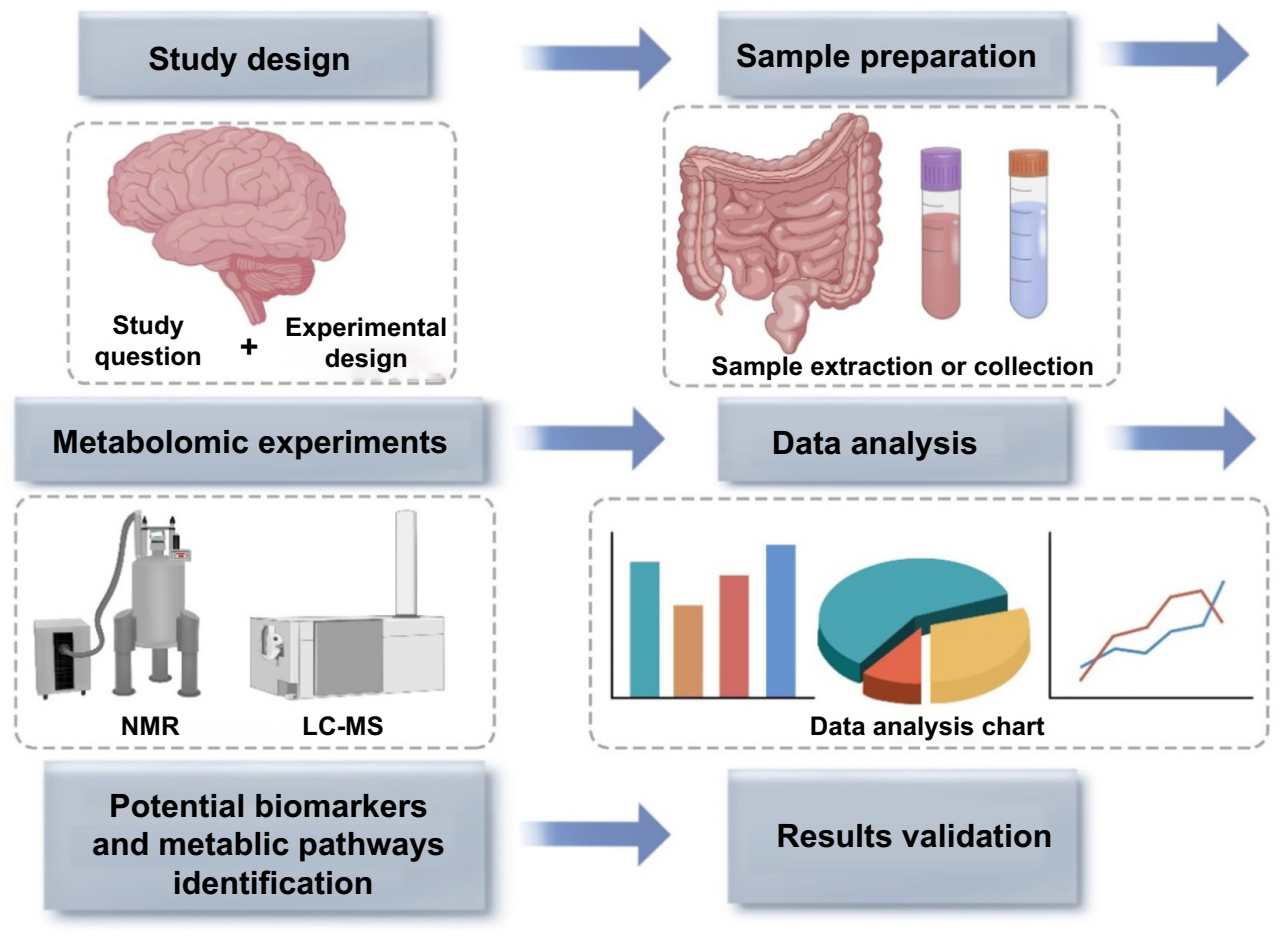
**The methodology of metabolomics.** NMR: Nuclear magnetic resonance; LC-MS: Liquid chromatography mass spectrometry.

**Figure 2.**
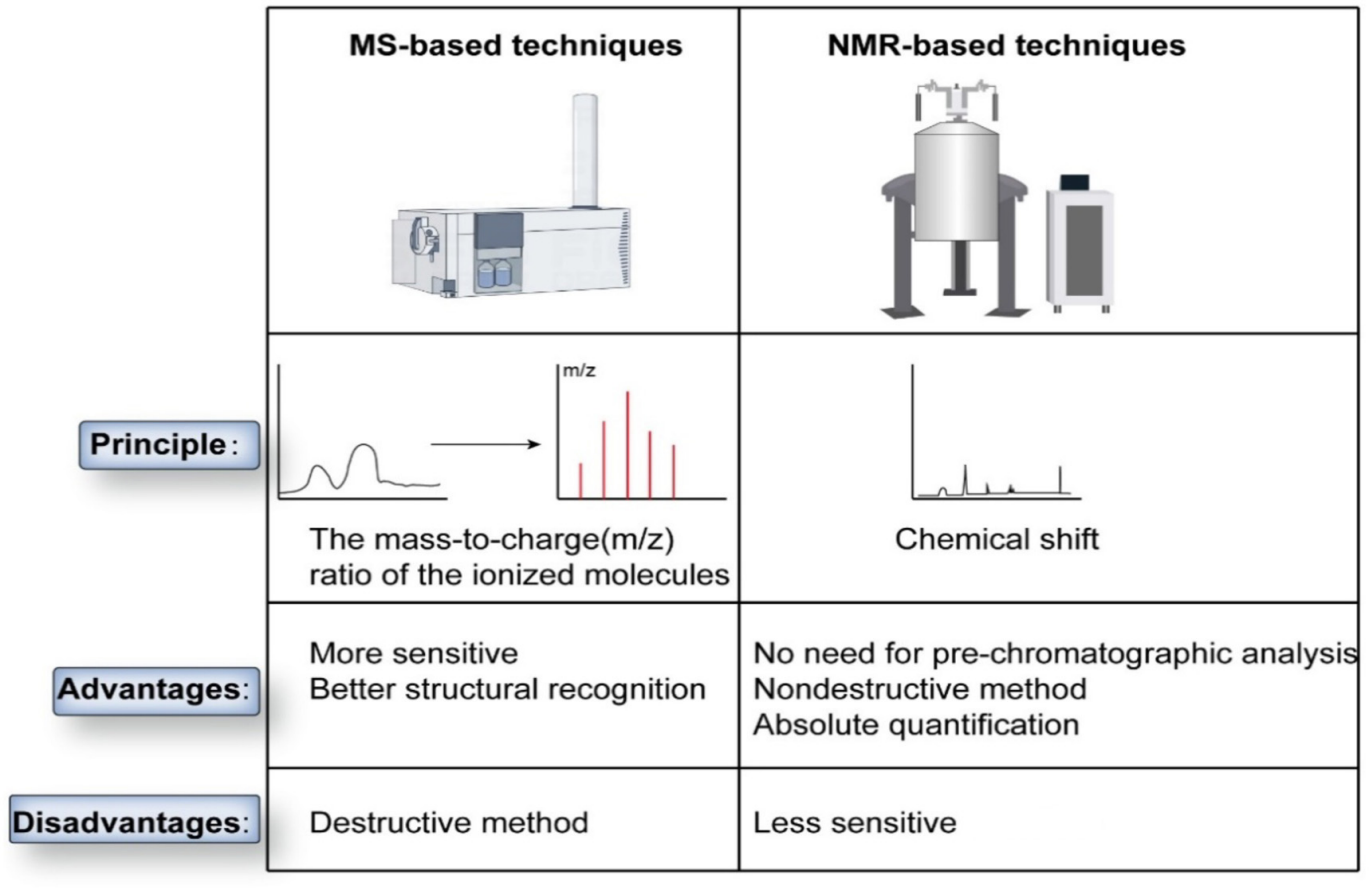
**The differences in the experimental methods currently used to assess metabolomics.** NMR: Nuclear magnetic resonance; MS: Mass spectrometry.

Each methodological framework manifests inherent merits. MS-based paradigms demonstrate elevated sensitivity and mandate diminutive specimen volumes but concomitantly induce specimen degradation [12]. Conversely, NMR-based paradigms are non-destructive albeit attenuated in sensitivity [11]. Both methodologies culminate in the generation of chromatograms or spectra, replete with salient peaks, each representing a singular compound in NMR or an ensemble of unique compounds in MS. Researchers subsequently engage expansive databases to correlate these peaks to specific chemical entities. Employing a diverse array of statistical algorithms, in conjunction with clustering and classification schemas, enables the discernment of potential biomarkers and metabolic pathways. The terminal phase encompasses the rigorous validation of these emergent findings (Figures 1 and 2) [13–17].

In the realm of modern metabolomics, investigations can be categorized into four primary modalities, namely, metabolite imaging, fluxomics, untargeted metabolomics, and targeted metabolomics [3]. The selection of a particular modality is dictated by both the instrumental capacities of the investigational laboratory and the specific scientific quandary to be addressed. Generally, targeted and untargeted metabolomics are the modalities most prevalently employed. Targeted metabolomics excels in the validation of hypotheses and the elucidation of biomarkers, principally leveraging LC-MS, GC-MS, and NMR methodologies for the precise identification and quantification of minuscule quantities of metabolites [18]. In contrast, untargeted metabolomics is tailored for the discovery of hitherto unidentified metabolites and the generation of novel hypotheses. This modality, compared to targeted metabolomics, offers extensive capabilities for metabolite characterization, often enabling the analysis of up to 10,000 distinct features through GC-MS, LC-MS, and CE-MS techniques [19]. Fluxomics, a specialized subset of targeted metabolomics, furnishes the capability to scrutinize the kinetics of metabolite reactions and monitor the transit of isotopic labels, employing LC-MS or NMR technologies [20]. Metabolite imaging serves as the fourth modality and facilitates the in vivo or in vitro detection and spatial visualization of metabolites within biological tissues [21, 22]. Pertaining to the sample matrices suitable for metabolomics analyses, a broad spectrum of biological samples can be utilized, including but not limited to cells, tissues, organs, biofluids, and whole organisms. To elucidate, while organs and tissues pose significant challenges in terms of extraction procedures, biofluids can be acquired noninvasively and serve as indicative proxies for organ-specific metabolic activities [11, 23, 24]. Regardless of the nature of the samples deployed, it is imperative to store them at −80^∘^C over extended durations to safeguard the integrity and thereby the veracity of the metabolomic experimental outcomes [3].

The application of metabolomics has transcended disciplinary boundaries, finding utility in a multitude of scientific domains such as botany, environmental science, toxicology, nutrition and food science, pharmaceutical R&D, and disease diagnostics, among others. Importantly, these fields exhibit substantive interrelations with human health outcomes, bolstering metabolomics as an invaluable tool for elucidating both physiological norms and pathological aberrations in myriad diseases [25, 26].

**Figure 3.**
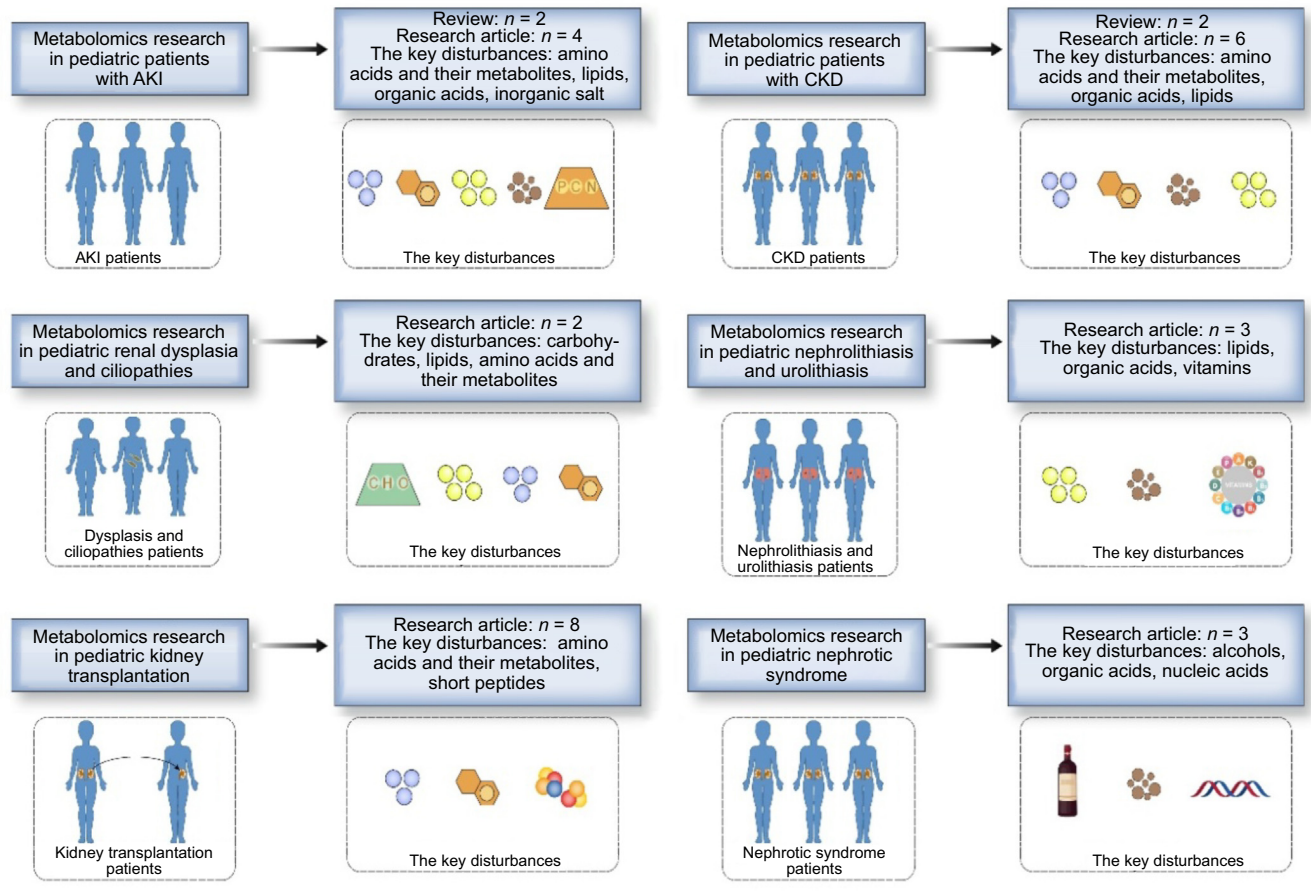
**A summary of the literature and the key disturbances in each disease subcategory.** AKI: Acute kidney injury; CKD: Chronic kidney disease.

In anatomical context, the kidney’s mitochondrial abundance is surpassed only by that of the heart, underscored by its role in reabsorbing in excess of 100 l of filtrate per diurnal cycle, thereby categorizing it as an organ with heightened energy demands. Elaborating further, kidneys execute a diverse array of metabolic functions orchestrated by heterogeneous cellular populations, which operate within a milieu of disparate oxygen tensions and osmotic conditions specific to the nephron [27]. Moreover, kidneys possess the aptitude to directly modulate circulating metabolite concentrations, via both uptake and release mechanisms. Subsequently, any decrement in renal function can swiftly propagate alterations in systemic metabolism. Given these attributes, metabolomics has been duly recognized as an exceptionally promising and efficacious methodology for research on renal pathologies [27, 28]. Current scientific endeavors have broadly incorporated metabolomics in the investigation of a multitude of renal diseases. A salient instance of the burgeoning interest in this domain is evidenced by a PubMed query for “pediatric kidney metabolomics,” which returned a mere two publications in January 2010, escalating to 220 by January 2023. This review aims to expound upon the advances in the metabolomic profiling of pediatric renal diseases over the past 12 years (Figure 3).

## Application of metabolomics in pediatric acute kidney injury

Acute kidney injury (AKI) is characterized by a precipitous diminution in glomerular filtration rate (GFR) within a constrained temporal window and is a prevalent complication in both pediatric and adult hospital admissions, with a noted ascendance in incidence rates [29]. According to a retrospective cohort study conducted in the United States, the incidence rate of AKI in the pediatric population was quantified as 3.9 cases per 1000 hospital admissions. Among these, a substantial proportion, ranging from 17.9% to 52%, manifested within Intensive Care Units (ICUs) or subsequent to the execution of corrective cardiac surgical procedures [29]. AKI has been empirically correlated with extended durations of mechanical ventilation, elevated mortality rates, and protracted hospital stays, with potential ramifications for long-term morbidity [30]. The definitional criteria for AKI, as promulgated by the Kidney Disease: Improving Global Outcomes (KDIGO), have gained widespread acceptance for the clinical staging of pediatric AKI. These criteria include a decrement in urine output to <0.5 mL/kg/h within a period ranging from 6 to 12 h, coupled with a serum creatinine (SCr) elevationof 50% or more [31]. However, SCr, though widely used, has been critiqued for its lack of sensitivity and reliability as an early indicator of altered renal function, principally because its concentration is unlikely to fluctuate until approximately 50% of renal function has been compromised [32]. This intrinsic latency renders SCr suboptimal as a diagnostic biomarker. The ideal biomarker would manifest alterations either at the stage of risk or upon initial detection of renal tissue damage, thereby facilitating early intervention prior to a decline in renal function [33].

Extant research has elucidated significant early fluctuations in several urinary and serum biomarkers correlated with AKI-induced renal cellular injury. Elevated concentrations of serum cystatin C (CysC), liver fatty acid-binding protein (L-FABP), kidney injury molecule-1 (KIM-1), and urinary neutrophil gelatinase-associated lipocalin (NGAL) have been authenticated as potential biomarkers for AKI [34]. Nonetheless, certain biomarkers may not be universally applicable across heterogeneous clinical settings or may exhibit inadequate specificity, particularly in relation to tubular injury. For instance, urinary NGAL concentrations may be elevated in the presence of an infection, without a correlative association with AKI incidence [33]. While the quest for novel and reliable early-stage biomarkers for AKI prediction remains a formidable challenge, incremental advancements have been realized in this avenue of investigation.

Over the preceding decade, innovative methodologies, such as metabolomics, have been instrumental in identifying novel putative biomarkers pertinent to AKI in pediatric cohorts (Table 1). Beger et al. undertook a comprehensive metabolomic analysis utilizing urine specimens from 40 pediatric patients subjected to cardiac surgery via cardiopulmonary bypass (CBP). Specifically, the post-operative development of AKI was observed in 21 patients within a temporal range of 48–72 h. Among the identified metabolites, omovanillic acid sulfate (HVA-SO4) emerged as a sensitive biomarker for AKI [35].

**Table 1:** Major findings of metabolomics research in pediatric patients with AKI

**References**	**Study design and methods**	**Population**	**Major findings**
Beger et al. [35]	Prospective, case-control study; UPLC/MS; MS/MS	21 children with AKI; 19 controls without AKI	Identification of urinary homovanillic acid sulfate (HVA-SO4) as a viable biomarker for AKI post-cardiac surgery in pediatric patients, displaying both sensitivity and predictive capabilities.
Wang et al. [36]	Prospective, case-control study; UPLC-QTOF/MS	27 septic children with AKI; 30 controls without AKI	Elucidation of a unique metabolic signature capable of differentiating pediatric patients with AKI from those without AKI in a septic context.
Muhle-Goll et al. [37]	Prospective, case-control study; NMR spectroscopy	65 children with AKI; 53 healthy children, and 31 critically ill children without AKI	Discovery of a diagnostic panel consisting of four metabolites (citrate, leucine, valine, and bile acid) that facilitate accurate AKI diagnosis.
Franiek et al. [33]	A re-analysis of two prospective cohort studies; GC-MS	28 children with AKI and 30 controls without AKI	Unveiling of a composite metabolite profile—encompassing 13 specific metabolites (PC.aa.C36.1, homovanillic acid, C7.DC, taurine, histidine, C2, methylmalonic acid, methionine, arginine, aspartic acid, C5.DC.C6.OH., Serotonin, acetylornithine )—that enables the prediction of AKI prior to clinical manifestation.

AKI: Acute kidney injury; UPLC: Ultra-performance liquid chromatography; MS: Mass spectrometry; GC-MS: Gas chromatography MS; UPLC/MS: Ultra-performance liquid chromatography mass spectrometry; MS/MS: Tandem mass spectrometry; UPLC-QTOF/MS: Ultra-performance liquid chromatography-quadrupole time-of-flight mass spectrometry.

In subsequent research, ultra-performance liquid chromatography-quadrupole time-of-flight MS (UPLC-QTOF/MS) was employed to scrutinize urine samples. A series of regression analyses were conducted to isolate promising biomarkers pertinent to sepsis-induced AKI. At the 12-h time point, the putative biomarkers included caprylic acid, trimethylamine N-oxide, DL-indole-3-lactic acid, and L-histidine. Furthermore, at the 24-h juncture, an additional cadre of four metabolites—3-methoxy-4-hydroxyphenylglycol sulfate, N4-acetylcytidine, 3-ureidopropionate, and gentisaldehyde—were delineated. The study posited that the assemblage of these metabolites serves as more efficacious diagnostic markers compared to their individual performance [36].

In 2020, Muhle-Goll et al. [37] promulgated their findings that a quartet of metabolites could serve as reliable diagnostic entities for AKI when evaluated via NMR spectroscopy (Table 1). Most recently, a metabolomic classifier was formulated that exhibited the capacity for preclinical identification of AKI risk within pediatric critical care settings. Researchers delineated a highly predictive profile, constituted by an amalgamation of 13 metabolites, which enabled the identification of at-risk patients approximately three days prior to the clinical manifestation of AKI. Subsequent investigations are mandated to independently corroborate these findings and to potentially augment the specificity of AKI predictors in alignment with the underlying etiological mechanisms [33].

## Application of metabolomics in pediatric chronic kidney diseases

Chronic kidney disease (CKD) constitutes a formidable public health quandary, characterized by irreversible renal damage that frequently culminates in renal dysfunction [38]. The ramifications of CKD and its progression to end-stage kidney disease (ESKD) during childhood are pervasive, adversely affecting life expectancy, growth, and overall development. Adults who have incurred kidney failure during childhood exhibit heightened susceptibility to cardiovascular diseases, infectious complications, and metabolic bone disorders, relative to the general populace [39]. Furthermore, the mortality rate for pediatric patients receiving renal replacement therapy is approximated to be 55 times greater than their counterparts in the general pediatric cohort [40].

Divergent from adult populations, the pediatric etiological landscape of CKD is predominantly influenced by congenital anomalies of the kidney and urinary tract, accounting for nearly 60% of all diagnoses and seldom accompanied by hypertension or proteinuria [41]. SCr, while ubiquitously utilized as a diagnostic marker for both AKI and CKD, lacks the requisite sensitivity and precision for early detection of CKD, thereby undermining preemptive intervention and mitigation strategies [42]. The advent of metabolomics offers a promising avenue for the elucidation of novel biomarkers that can facilitate early diagnosis and enhance prognostic outcomes for pediatric CKD patients.

Benito et al. executed a prospective cohort-based study, revealing that five metabolites exhibited elevation independent of SCr levels (Table 2), while dimethylglycine levels ascended in CKD patients with SCr concentrations exceeding 12 mg/mL. Further investigations across broader populations are imperative for confirming the utility of these metabolites as viable predictors of pediatric renal function [38]. Subsequently, they disseminated an additional publication, employing untargeted metabolomics for the discovery of plasma biomarkers suitable for early CKD diagnosis in the pediatric cohort. Four metabolites were identified to be elevated in CKD-afflicted patients (Table 2), while bilirubin levels demonstrated a significant decline, necessitating further validation and corroborative studies [43].

**Table 2:** Major findings of metabolomics research in pediatric patients with CKD

**References**	**Study design and methods**	**Population**	**Major findings**
Benito et al. [38]	Prospective cohort-based study; LC-QTOF-MS	32 patients diagnosed with CKD across varying stages; Control group of 24 ostensibly healthy subjects	Identification of five metabolites (glycine, citrulline, creatinine, ADMA, and SDMA) exhibiting elevated levels independent of serum creatinine (SCr); Increment in dimethylglycine observed when SCr levels surpassed 12 mg/mL.
Benito et al. [43]	Prospective cohort-based study; LC-QTOF-MS	32 patients manifesting CKD at distinct stages; 26 healthy control subjects	Ascertainment of four augmented metabolites in CKD-afflicted patients (sphingosine-1-phosphate, n-butyrylcarnitine, cis-4-decenoylc arnitine, and an unidentified feature with m/z 126.0930); Concurrent significant reduction in bilirubin levels.
Sood et al. [44]	Population-level cohort-based study; Metabolic profiling of neonates from 2006 to 2015 for the detection of metabolic profiles at birth possibly related to higher CKD or dialysis risk	1,288,905 newborns with accessible newborn screening data (2086 developed CKD, 641 required dialysis)	Strongest correlations observed between amino acids and acylcarnitines to endocrine markers (e.g., 17-hydroxyprogesterone), acylcarnitine ratios, and specific amino acid ratios for CKD; Amino acid ratios (phenylalanine/glycine, phenylalanine/tyrosine, citrulline/tyrosine), acylcarnitine ratios, and ratio of amino acids to acylcarnitine strongly correlated for dialysis.
Brooks et al. [45]	Prospective cohort-based study; targeted metabolomics for plasma of adolescents with mild to moderate CKD (stages 2 and 3b)	40 patients sub-divided into two cohorts based on age, gender, and CKD etiology	Delineation of five discernibly altered metabolites (acylcarnitine, creatinine, Kyn, Trp, phosphatidylcholine) and six salient metabolic ratios (SDMA/ADMA, Phe/Trp, Pro/Cit, Kyn/Trp, Orn/Cit, Tyr/Cr) between the cohorts.
Denburg et al. [39]	Multicenter prospective cohort-based study; Untargeted metabolomics to ascertain novel metabolite correlations with CKD progression in pediatric patients who did not rely on developed clinical predictors and highlight the roles played by biologic pathways selected	645 participants with eGFR of 30–90 mL/min per 1.73 m^2^	For eGFR > 60 mL/min per 1.73 m^2^, seven metabolites, namely gulonate, 2-methylcitrate/homocitrate, lanthionine, C-glycosyltryptophan, pesudouridine, 5,6-dihydrouridine, and N6-carbamoylthreonyladenosine, exhibited significant relationships with CKD progression. For eGFR < 60 mL/min per 1.73 m^2^, elevated levels of tetrahydrocortisol sulfate correlated with a reduced risk of CKD progression.

CKD: Chronic kidney disease; LC-QTOF-MS: Liquid chromatography-quadrupole time-of-flight mass spectrometry; eGFR: Estimated glomelural filtration rate; m/z: Mass-to-charge ratio.

Concurrently, population-level cohort studies have yielded metabolites that exhibit strong associations with CKD or dialysis [44] (Table 2). Brooks et al. [45] conducted a prospective cohort-based study, identifying discrepancies in five metabolites and six ratios between adolescents with mild-to-moderate CKD (stages 2 and 3b) (Table 2). In 2021, Denburg et al. promulgated findings from a multicenter prospective cohort study, revealing that among children with an estimated GFR (eGFR) exceeding 60 mL/min per 1.73 m^2^, seven metabolites exhibited robust correlations with CKD progression (Table 2). Conversely, elevated levels of tetrahydrocortisol sulfate were inversely associated with CKD risk in individuals with eGFR below 60 mL/min per 1.73 m^2^ [39].

## Application of metabolomics in pediatric renal dysplasia and ciliopathies

The manifestation of renal dysplasia serves as a seminal etiological factor contributing to CKD and ultimately, ESKD during both childhood and early adulthood. This pathological construct is frequently delineated in pediatric cohorts necessitating renal replacement therapy. Ultrasonographic evaluations afford clinicians the ability to diagnose renal dysplasia, revealing parameters, such as kidney size (either normative or diminutive), augmented echogenicity, and deficient or ineffectual corticomedullary differentiation, often concomitant with the presence of diminutive cystic structures. The paucity of early diagnostic markers, attributed to the asymptomatic nature of renal dysplasia in its nascent stages, imposes limitations on preemptive therapeutic interventions. Given the looming risk of ESKD, the exigency for early diagnostic measures is underscored. Macioszek et al., in their 2021 publication, explored urinary metabolomic signatures, contrasting children with renal dysplasia against healthy controls. Perturbations in key biochemical pathways were discerned, including those governing glycolytic activity, lipid metabolism, purine and amino acid turnover, and both the tricarboxylic acid (TCA) and urea cycles. A cohort of 28 metabolites evinced statistically significant divergences, nine of which exhibited discriminative capacity in stratifying patients based on eGFR [46] (Table 3).

**Table 3:** Major findings of metabolomics research in pediatric renal dysplasia and ciliopathies

**References**	**Study design and methods**	**Population**	**Major findings**
Macioszek et al. [46]	Prospective, case-control study; GC-QQQ/MS, LC-TOF-MS	39 pediatric patients subjected to renal dysplasia; 33 healthy children as controls	Discovery of 28 metabolites manifesting discernible disparities between pediatric patients afflicted with renal dysplasia and the healthy control cohort; Nine among these metabolites were elucidated as significant for differentiating subjects having reduced and normal eGFR.
Baliga et al. [48]	Randomized, double-blind, placebo-controlled stage 3 clinical trial; HPLC–MS/MS	31 pediatric patients administrated using pravastatin; 27 controls with placebo	Identification of 37 metabolites that exhibited promising characteristics for distinguishing plasma profiles in children diagnosed with ADPKD from those in healthy controls.

MS: Mass spectrometry; ADPKD: Autosomal dominant polycystic kidney disease; eGFR: Estimated glomelural filtration rate; GC-QQQ/MS: Gas chromatography coupled to triple quadrupole mass spectrometry; LC-TOF-MS: Liquid chromatography coupled to time-of-flight mass spectrometry; HPLC-MS/MS: High-performance liquid chromatography-tandem mass spectrometry; ADPKD: Autosomal dominant polycystic kidney disease.

**Table 4:** Major findings of metabolomics research in pediatric nephrolithiasis and urolithiasis

**References**	**Study design and methods**	**Population**	**Major findings**
Wen et al. [56]	Prospective study; UPLC-MS	30 patients with kidney stones; 20 normal controls	A constellation of 40 metabolites was delineated as significantly perturbed in subjects harboring urolithiasis, predominantly implicating pathways such as retinol metabolism, steroid hormone biosynthesis, and porphyrin and chlorophyll metabolism.
Greed et al. [57]	Case reports; tandem mass spectrometry	2 pediatric cases with kidney stones	Utilization of tandem mass spectrometry for urine screening was elucidated as an expedient, high-throughput modality capable of detecting cases of primary hyperoxaluria type 3 (PH3).
Denburg et al. [49]	A matched case-control study; shotgun metagenomic sequencing and untargeted metabolomics on stool samples	44 pediatric patients with kidney stones containing > 50% calcium oxalate and 44 age-, sex-, and race-matched controls	Depletion of butyrate-producing (Roseburia and Clostridium species) and oxalate-degrading (Enterococcus faecalis, Enterococcus faecium, and Bifidobacterium animalis) gut microbiota emerged as potential antecedent determinants of early-onset calcium oxalate kidney stones; the nadir of microbial diversity was observed in individual patients who were diagnosed with stones for the inaugural time, falling within an age bracket of 9–14 years.

UPLC-MS: Ultra-performance liquid chromatography mass spectrometry.

Ciliopathies encapsulate a pantheon of pathologies engendered by aberrant ciliary function, exerting multisystemic impact with a particular propensity for hepatic and renal sequelae. Autosomal recessive polycystic kidney disease (ARPKD) and autosomal dominant polycystic kidney disease (ADPKD) are categorically identified as the predominant ciliopathic entities. Furthermore, myriad cilia-associated genetic loci have been implicated in an array of human maladies, inclusive of Nephronophthisis, Bardet–Biedl Syndrome, Alstrom Syndrome, and Joubert Syndrome [47].

Baliga et al. embarked on a comparative analysis of metabolomic profiles in plasma samples obtained from pediatric ADPKD patients and healthy controls, with an aim to unearth potential biomarkers and molecular pathways germane to disease progression. The study delineated salient associations between metabolites from asparagine metabolic pathways, namely, urea and components of the methylation cycle, as well as arginine, glutamine, and tryptophan, with the ontogenesis and advancement of ADPKD. These identified metabolic pathways emerge as putative therapeutic targets, necessitating exhaustive scrutiny to evaluate their potential utility in the early amelioration of ADPKD. Among the 95 metabolites delineated, 37 manifested significant discriminative potential in differentiating pediatric ADPKD patients from healthy subjects [48] (Table 3).

## Application of metabolomics in pediatric nephrolithiasis and urolithiasis

The phenomena of urolithiasis—stone formation within the urinary tract—and nephrolithiasis—renal lithogenesis—have garnered considerable attention due to their rising incidence in pediatric and adolescent populations [49]. These conditions are demonstrably correlated with a spectrum of adverse health outcomes, including but not limited to, osseous fractures, compromised bone mineral density, renal functional decline, and an augmented risk of cardiovascular pathologies [50–52]. Intriguingly, the incidence rates of nephrolithiasis and urolithiasis appear disproportionately elevated in younger cohorts. Notably, pediatric and adolescent populations manifest a higher predilection for stone recurrence compared to their adult counterparts [53, 54]. This shift toward a younger age of onset remains an enigmatic clinical observation, albeit conjectural links to dietary habits and environmental antibiotic exposures have been postulated [55].

Wen et al. [56] identified 40 distinct metabolites that were significantly dysregulated in patients with urolithiasis, thus laying the groundwork for novel preventive and therapeutic interventions (Table 4). In a seminal study by Greed et al., urinary metabolites from two pediatric cases of renal calculi were scrutinized utilizing tandem MS. Both cases exhibited markedly elevated levels of 4-hydroxyglutamate [4OHGlu, a quintessential marker for primary hyperoxaluria type 3 (PH3)] and affiliated metabolites. The identification of bi-allelic deleterious mutations in the *HOGA1* gene corroborated the diagnosis of PH3. The investigators posited that 4OHGlu ought to be integrated into metabolomic panels aimed at screening patients with nebulous etiologies of nephrolithiasis [57].

In a pivotal research endeavor, Denburg et al. explored the nexus between early-onset calcium oxalate kidney stone disease and the composition and functionalities of the gut microbiota. Compared to a control cohort, patients with renal lithiasis manifested a paucity of microbial diversity. Amongst 31 less abundant taxa, seven were identified as butyrate producers, while three were implicated in oxalate degradation (Table 4). The diminution of butyrate-producing and oxalate-degrading bacterial taxa was linked to disturbances in the host metabolome, ostensibly serving as antecedent determinants for early-onset calcium oxalate kidney stones. An additional revelation from the study was the age-dependent variance in microbial diversity, which was particularly attenuated in patients who manifested renal lithiasis between the ages of 9 and 14 [49].

## Application of metabolomics in pediatric kidney transplantation

Kidney transplantation (KTx) has emerged as the modality of choice for ESKD management [58]. Given the scarcity of both available renal allografts and willing donors [59], optimizing the longevity of the transplanted organ remains a clinical imperative. While short-term graft survival has indeed witnessed appreciable enhancement, courtesy of advances in organ procurement strategies, surgical techniques, and immunosuppressive pharmacotherapy [60], long-term graft outcomes have remained recalcitrant to improvement [61]. Current diagnostic modalities, such as SCr and proteinuria, are characterized by suboptimal sensitivity and specificity [62]. Moreover, renal biopsy, though considered the gold standard, suffers from invasiveness and restricted feasibility, particularly within pediatric cohorts [63]. Consequently, there is an unmet clinical need for the identification of novel, sensitive, and specific biomarkers pertinent to KTx.

Blydt-Hansen et al. embarked upon a prospective investigation to evaluate the utility of urinary metabolomics for noninvasively diagnosing T-cell-mediated rejection (TCMR) in pediatric KTx recipients. They delineated a urinary metabolite profile indicative of TCMR risk. Interestingly, 5–10 of the identified metabolites were congruent with borderline tubulitis, thereby hinting at a continuum of allograft injury associated with TCMR. The findings corroborate the superior sensitivity, specificity, and noninvasiveness of urinary metabolomics vis-à-vis traditional SCr-based diagnostic paradigms [64]. In a subsequent prospective study, Blydt-Hansen et al. [65] discerned a unique urinary metabolic classifier for antibody-mediated rejection (AMR), pending serial validation for clinical implementation.

In a pediatric cohort-centric study, Archdekin et al. [66] validated the potential utility of a urinary metabolite classifier for the efficacious differentiation of non-rejection kidney injury (NRKI) from rejection, without the necessity for invasive procedures. A recent endeavor by Sigdel et al. [67] underscored that targeted metabolomic analyses of urine samples, congruent with biopsy findings, are proficient in the noninvasive detection of diverse graft injury phenotypes, engendering elevated diagnostic confidence.

Collectively, the extant body of metabolomic research in the domain of KTx lends substantive credence to the paradigm-shifting role of urinary metabolites as non-invasive diagnostic biomarkers for both rejection and NRKI. These advancements significantly augment personalized medical interventions, thereby optimizing patient care management through the exploitation of individual molecular risk stratification.

## Application of metabolomics in pediatric nephrotic syndrome

Nephrotic syndrome (NS) is characterized by a constellation of clinical features: pronounced albuminuria, hypercholesterolemia, hypoalbuminemia, and edema. This syndrome emanates from altered pathophysiological underpinnings, primarily associated with enhanced permeability of glomerular filtration membranes to plasma proteins, culminating in substantial urinary loss of albumin [68]. Epidemiological data delineate a pediatric incidence of NS ranging from 2 to 7 cases per 100,000 children annually [69, 70]. Glucocorticoid therapy (GC), despite being a mainstay for over six decades, continues to be the pivotal treatment modality. Based on the responsiveness to GC, NS is categorized into steroid-sensitive NS (SSNS) and steroid-resistant NS (SRNS). The development of early diagnostic biomarkers capable of prognosticating NS and its therapeutic responsiveness in pediatric patients remains inchoate. The absence of prompt diagnosis and intervention can result in recurrence rates as high as 40% post maturation into adulthood, with a potential progression to ESKD [71, 72]. Inefficacious long-term GC therapy exposes some patients to an elevated risk of disease progression and associated adverse effects.

In a seminal 2021 study, Guo et al. [73] delineated 12 differential serum metabolites with promising diagnostic potential for pediatric NS, employing targeted and non-targeted metabolomic techniques (GC-MS and UPLC-MS). A subsequent case-control investigation discerned seven metabolites (α-KG, bilirubin, NAD, NADPH, D-sorbitol, dulcitol, and D-mannitol) as plausible biomarkers for risk stratification and early diagnosis of SSNS in pediatric cohorts [74]. These investigations collectively herald an evolving paradigm for monitoring the pathophysiological trajectory of pediatric NS and elucidating associated metabolic perturbations. Gooding et al. identified three metabolites (creatinine, glutamine, and malonate) via 1H NMR metabolomic analyses as prospective biomarkers for assessing SRNS at disease onset. Additional candidate biomarkers have been unearthed to augment our comprehension of molecular pathways potentially governing clinical steroid resistance [75].

Concomitant with advancements in NS, metabolomics has also gained traction in the exploration of vesicoureteral reflux [76], IgA vasculitis with nephritis [77], and nephropathic cystinosis [84] in pediatric populations over the past decade. Despite these strides, several limitations persist. Numerous metabolites have been reported in both serum and urine specimens; however, subsequent validation is requisite to ascertain their status as bona fide markers of pathogenesis vs mere epiphenomena of metabolic dysfunction engendered by kidney disease. The 7unequivocal identification of metabolomic signatures remains an elusive goal for the scientific community [34].

## Integration of multi-omics: Current applications for future directions in pediatric nephrology

Omics encompasses genomics, transcriptomics, proteomics, and metabolomics, collectively serving as an emergent avenue that harnesses systems biology methodologies to illuminate complex biological systems. This multi-disciplinary framework provides an invaluable platform for scrutinizing molecular mechanisms, identifying robust biomarkers, and proffering novel therapeutic strategies for pediatric renal pathologies heretofore unavailable [79]. Multi-omics integration holds the potential to deliver nuanced insights into pediatric kidney diseases by synergizing data from disparate biological levels. Notwithstanding its promise, the seamless amalgamation and analysis of voluminous and intricate omics datasets present considerable challenges; conventional analytical tools designed for singular omic platforms are generally inapplicable for multi-omic data integration [79]. The advent of advanced bioinformatic algorithms—such as MetaboAnalyst 5.0, multi-omic factor analysis (MOFA), mixOmics, and similarity network fusion (SNF)—has substantially enhanced data processing capabilities [80–83]. Furthermore, the confluence of machine learning (ML) and deep learning (DL) methodologies with exhaustive analyses promises to revolutionize precision medicine, enable sophisticated patient stratification, and refine disease prognostication [84]. The judicious selection of analytical instruments and acquisition of relevant technical expertise are integral and assume paramount importance in effectively addressing the intricate biological queries posited.

## Conclusion

The intrinsic heterogeneity of pediatric renal diseases poses a formidable challenge for clinicians in the field of pediatric nephrology, often culminating in delayed or advanced-stage diagnoses. Renal function exerts multifaceted influences on circulating metabolite levels, which reciprocally exhibit diverse functional roles across various physiological systems. Research in metabolomics has augmented our grasp of this intricate landscape and holds the potential to further expound upon metabolic interactions across a gamut of organ systems. Nevertheless, additional empirical inquiries are indispensable for transmuting these nascent discoveries into tangible advancements in the diagnostic, prognostic, and therapeutic paradigms of clinical renal diseases.
